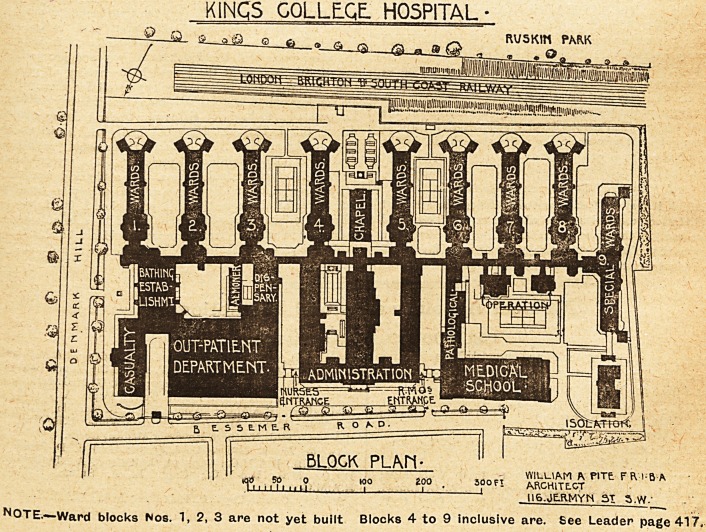# Londoners' Hospital Beds Threatened

**Published:** 1919-02-15

**Authors:** 


### The Hospital

The Workers' Newspaper of Administrative Medicine and Institutional Life, Administration, National Insurance and Health.

No. 1706, Vol. LXV. SATURDAY, FEBRUARY 15, 1919. PRICE TWOPENCE

### LONDONERS' HOSPITAL BEDS THREATENED.

The Metropolis of the Empire has long been insufficiently supplied with hospital beds, if the needs of all classes of Londoners are to be adequately met. Taking Inner London in 1916 first, the total average available beds in 1,962 voluntary hospitals was 14,203, occupied by 155,403 in-patients, of which 11,741 beds were on an average occupied daily. This is equal to a provision of 3.14 beds per thousand of the population within this area. If we add Outer London and include the inhabitants in places within the Metropolitan and City Police Districts—that is, within a radius of 15 miles from Charing Cross—the number of beds would be but 1.95 beds per thousand of the population. A modern, up-to-date hospital system such as will supply speedily the requirements of all classes of the population, must provide at least twice as many beds as those now available. Even so there will probably be found a growing demand for still further hospital beds, for the use of members of the middle and upper classes, in addition to some of the working-classes, who will desire to pay some portion, if not the whole, of the cost of their hospital treatment.

It is notorious that one great drawback to the present hospital provision for Londoners arises from the fact that several of the hospitals are too centralised, whereas the tendency for many years has been for the population to move out from Central London to Outer London, and farther still. The population of Inner London is now, according to the latest available census returns, 4,521,301. But the population of Outer London has increased greatly, and the total population resident within a radius of fifteen miles from Charing Cross approximates to-day 7,251,000, say, seven and a quarter millions. Further hospital provision must be forthcoming quickly, because since the war the civil population has had to stand aside, and the waiting lists, if they have been kept up, will reveal an army of suffering Londoners of both sexes and all ages which might indeed startle the public. In a line the number of hospital beds required in London is at least double that at present provided.

The policy of taking the hospital from the centre to the population in the outer ring was notably adopted by the authorities of King's College Hospital when the present Lord Hambleden in 1904, as chairman of the Hospital Board and the Removal Fund Committee, acquired and presented a site of just over twelve acres in south-east London. It is situated on Denmark Hill, and is adjacent to Ruskin Park, containing twenty-two acres, from which it is divided by the South-Eastern and Chatham, and the London, Brighton and South Coast Railways. The proportions of unoccupied land to buildings is: land 7.75 acres, buildings 4.50 acres. The hospital is divided into two sections by the main hospital corridor—that is, into a northern and southern portion. The ward pavilions of which all are built except Nos. 1, 2, and 3, occupy the whole of the southern portion of the site stretching from east to west in an unbroken line. There are other buildings in contemplation to go on this site, which are not shown on the block plan. The ward pavilions occupy the best aspect which the site affords, being ends-on to the outlook over Ruskin Park. From the first the criticism was raised whether the buildings which were ultimately to be placed upon the site of twelve acres would not crowd it? This criticism has been given greater, rather than less, force by hygienic developments, and the growth of hospital opinion, since the site was purchased. Its force can be judged by reference to page 427 of our present issue, where we give the block plan with most of the proposed buildings marked upon it.

Some ten years later the late Sir John Wolfe Barry, K.C.B., F.R.S., chairman of Westminster Hospital, had to face the clear necessity which had arisen to remove the Westminster Hospital to a new site. After devoting much time and hard work to the solution of the problem thus presented, Sir John Wolfe Barry formulated a very able scheme to transfer Westminster Hospital to an excellent site on Clapham Common. Of this he acquired the freehold, where a hospital could supply the needs of some 400,000 people who have long been in serious want of one in their midst. The scheme was undoubtedly one which would entitle the hospital authorities to the sympathy of all intelligent

Londoners, and in our opinion promptly secure for the Building Fund all the money needed to defray the cost of a modern hospital of the highest type with an efficient medical school. It would prove an inestimable boon to Londoners, for the site at Clapham is an island site, with wide roads adjoining, having a southerly aspect over Clapham, and a northerly view over London to Hampstead. It adjoins the crowded and rapidly growing districts of Nine Elms, Stockwell, Battersea, Clapham, Brixton, Wandsworth, Balham and Tooting, where exists a population of some 400,000 people. This makes it of interest to all generous supporters of hospitals within the Metropolis, and secures, we have no doubt, that the money which may be required to perfect and maintain it will be readily and gladly supplied directly things settle themselves a little after the war, and the Governors of the Westminster Hospital issue their appeal. There is a rumour that seven members out of a House Committee of thirty-five, exclusive of ex-officio members, have summoned a meeting of the Governors of Westminster Hospital, in the endeavour to throw over Sir Wolfe Barry's able scheme. An overwhelming majority, five to one, of the Committee have, as we understand, properly expressed themselves in favour of the Clapham site, and declared that the four objections advanced against it have no foundation in fact. It must be clear to our readers and to the hospital world generally that to sink the Westminster Hospital, its traditions and identity, in another institution would be to perpetrate a wanton injustice on the citizens of London. It might indeed be followed by disastrous financial results to the important hospital at Denmark Hill, which has been mentioned in this connection.

Is there a wealthy man of business, interested in hospitals, who would give his money to enable seven governors of one old hospital to destroy and sink its individuality and reputation, to secure a damaging blow to our latest and best type of general hospital, and to deprive 400,000 poor Londoners of the hospital accommodation which they urgently need and would otherwise rapidly secure? Can it be that the present medical staff of Westminster Hospital, or some of them, feel they are not strong enough themselves to run a new and up-to-date hospital? If so, why are they not men enough to say so and make room for better men, of whom many, full of up-to-date experience, will soon be returning from the various Fronts?

**NOTE.—Ward blocks Nos. 1, 2, 3 are not yet built Blocks 4 to 9 inclusive are.** **See Leader page 417.**

## Figures and Tables

**Figure f1:**